# Supplementary material for: Novel Insights and Genomic Characterization of Coral-Associated Microorganisms from Maldives Displaying Antimicrobial, Antioxidant, and UV-Protectant Activities
Source: Biology (Basel). 2025 Apr 11;14(4):401. doi: 10.3390/biology14040401 (PMC12024893; doi:10.3390/biology14040401)
Supplement: Supplementary file 1 [file biology-14-00401-s001.zip › biology-3544427-supplementary.pdf]

Supplementary Results

Table S1. Percentages of relative abundances of families for microbiomes of *Porites lobata, Acropora gemmifera*, water and samples. Family composition is reported for corals, water and sediments sampled in two sampling depths (5m, shallow; 17 m, deep). "large" refers to colonies ≥ 30 cm in diameter, "small" refers to colonies ≤ 10 cm in diameter.

	P. lobata				***A. gemmifera***				**water**		**sediment**	
**Taxonomy**	shallow		deep		shallow		deep		shallow	deep	shallow	deep
	**large**	**small**	**large**	**small**	**large**	**small**	**large**	**small**				
Others	17,9	20,7	28,9	31,1	35,2	12,4	98,3	94,4	40,6	35,8	37,7	40,6
PAUC26f	0,0	0,0	0,0	5,4	0,0	0,0	0,0	0,0	0,1	0,1	0,0	0,4
Hyphomicrobiaceae	0,0	0,0	1,0	0,0	0,0	0,0	0,0	0,0	2,3	0,9	0,7	1,6
Vibrionaceae	0,0	0,6	0,2	0,8	2,9	0,1	0,2	0,1	0,4	0,6	0,7	0,2
Ilumatobacteraceae	0,0	0,0	0,1	0,0	0,0	0,0	0,0	0,0	1,3	3,3	1,7	0,6
Methyloligellaceae	0,0	0,2	1,7	0,0	0,0	0,0	0,0	0,0	1,7	1,1	0,6	1,9
SAR202_clade	0,0	0,0	0,0	7,2	0,0	0,0	0,0	0,0	0,0	0,0	0,0	0,1
Stappiaceae	4,9	0,7	0,3	0,1	0,0	0,0	0,0	0,0	0,8	0,4	0,2	0,0
Thermoanaerobaculaceae	0,0	0,1	0,4	0,1	0,0	0,0	0,0	0,0	3,2	0,5	0,4	2,7
Nitrospiraceae	0,0	0,0	0,0	3,6	0,0	0,0	0,0	0,0	1,0	0,1	0,1	2,7
Ruminococcaceae	8,9	0,0	0,0	0,0	0,0	0,0	0,0	0,0	0,0	0,0	0,0	0,0
Desulfocapsaceae	0,0	0,0	0,0	0,0	0,0	0,0	0,0	0,0	1,0	3,6	3,8	0,6
KI89A_clade	0,0	0,0	0,2	3,3	0,0	0,0	0,0	0,0	1,7	1,3	1,5	1,1
Alteromonadaceae	0,0	5,2	0,0	0,4	0,2	0,0	0,1	0,1	0,0	1,5	1,5	0,0
Halieaceae	0,0	0,1	0,2	0,0	0,0	0,0	0,0	0,0	1,6	4,7	4,1	0,7
Flavobacteriaceae	0,2	2,0	0,9	0,2	0,0	0,0	0,5	0,1	0,6	3,6	4,6	0,0

BD2-11_terrestrial_group	0,0	0,1	0,4	7,3	0,0	0,0	0,0	0,0	1,7	1,0	1,2	2,0
Desulfosarcinaceae	0,0	0,0	0,0	0,0	0,0	0,0	0,0	0,0	5,1	2,2	2,3	4,8
Nitrosococcaceae	0,1	0,1	0,1	6,6	0,0	0,0	0,0	0,0	2,8	1,0	1,6	3,5
Spirochaetaceae	8,3	0,4	0,1	3,2	0,0	0,0	0,0	0,0	0,8	2,7	2,4	1,5
Microbacteriaceae	0,0	0,0	19,7	0,0	0,0	0,0	0,0	0,0	0,0	0,0	0,0	0,0
Cellvibrionaceae	0,0	20,8	0,2	0,0	0,0	0,0	0,0	0,0	0,3	0,0	0,1	0,0
Desulfovibrionaceae	28,3	0,1	0,0	0,0	0,0	0,0	0,0	0,0	0,0	0,3	0,4	0,0
NB1-j	0,0	0,0	0,4	0,0	0,0	0,0	0,0	0,0	9,1	4,1	4,1	13,1
Woeseiaceae	0,0	0,2	0,9	4,5	0,0	0,0	0,0	0,0	7,3	5,9	6,9	5,7
Kiloniellaceae	0,1	0,7	3,0	1,4	0,0	0,0	0,0	0,0	7,9	4,3	5,6	9,7
Microtrichaceae	0,0	0,1	0,4	22,3	0,0	0,0	0,0	0,0	2,9	1,7	1,7	4,1
Cyclobacteriaceae	28,7	0,3	0,2	0,0	0,1	0,0	0,0	0,0	1,7	4,4	4,8	0,6
Rhodobacteraceae	1,1	9,7	1,8	2,2	0,0	0,0	0,1	0,1	2,4	13,0	9,8	0,9
Rhizobiaceae	1,4	3,0	38,2	0,2	0,0	0,0	0,0	0,0	1,8	2,0	1,5	1,1
Endozoicomonadaceae	0,1	34,4	0,7	0,0	61,7	87,5	0,7	5,2	0,0	0,0	0,0	0,0

Table S2. Description of the function of oxidative and nitrogen stress-related genes (ROS and RNS) present in the 39, 79, 92 and 93 genomes by RAST annotation.

	Strain	contig_id	Start	Stop	Strand	Function
	39	Scaffold_10	296315	296758	+	Hypothetical flavoprotein YqcA (clustered with tRNA pseudouridine synthase C)
	39	Scaffold_10	334440	336017	+	Glutamate--cysteine ligase (EC 6.3.2.2)
	39	Scaffold_14	495623	493881	-	Gamma-glutamyltranspeptidase (EC 2.3.2.2) @ Glutathione hydrolase (EC 3.4.19.13)
	39	Scaffold_4	22891	21131	-	Gamma-glutamyltranspeptidase (EC 2.3.2.2) @ Glutathione hydrolase (EC 3.4.19.13)
	39	Scaffold_8	286662	287621	+	Glutathione synthetase (EC 6.3.2.3)
	39	Scaffold_8	608774	607080	-	Gamma-glutamyltranspeptidase (EC 2.3.2.2) @ Glutathione hydrolase (EC 3.4.19.13)
	79	Scaffold_11	52288	50792	-	hypothetical protein
**Glutathione:_biosynthesis**	79	Scaffold_14	1566	817	-	Regulatory protein RecX
	79	Scaffold_7	163792	162476	-	Glutamate--cysteine ligase (EC 6.3.2.2)
	79	Scaffold_8	521170	519353	-	Gamma-glutamyltranspeptidase (EC 2.3.2.2) @ Glutathione hydrolase (EC 3.4.19.13)
	92	Scaffold_1	730143	731132	+	Integral membrane protein
	92	Scaffold_1	123773 2	123951 9	+	Gamma-glutamyltranspeptidase (EC 2.3.2.2) @ Glutathione hydrolase (EC 3.4.19.13)
	92	Scaffold_2	757206	758054	+	Oxidoreductase
	93	Scaffold_1	197001	195061	-	Gamma-glutamyltranspeptidase (EC 2.3.2.2) @ Glutathione hydrolase (EC 3.4.19.13)
	93	Scaffold_9	555924	557159	+	Glutamate--cysteine ligase (EC 6.3.2.2)
	39	Scaffold_10	334440	336017	+	Glutamate--cysteine ligase (EC 6.3.2.2)
**glutamate--cysteine (gshA)**	79	Scaffold_7	163792	162476	-	Glutamate--cysteine ligase (EC 6.3.2.2)
	93	Scaffold_9	555924	557159	+	Glutamate--cysteine ligase (EC 6.3.2.2)
**gamma-glutamyl_cycle**	39	Scaffold_10	296315	296758	+	Hypothetical flavoprotein YqcA (clustered with tRNA pseudouridine synthase C)
	39	Scaffold_10	336134	336742	+	putative lipoprotein

	39	Scaffold_14	495623	493881	-	Gamma-glutamyltranspeptidase (EC 2.3.2.2) @ Glutathione hydrolase (EC 3.4.19.13)
	39	Scaffold_4	22891	21131	-	Gamma-glutamyltranspeptidase (EC 2.3.2.2) @ Glutathione hydrolase (EC 3.4.19.13)
	39	Scaffold_8	286662	287621	+	Glutathione synthetase (EC 6.3.2.3)
	39	Scaffold_8	608774	607080	-	Gamma-glutamyltranspeptidase (EC 2.3.2.2) @ Glutathione hydrolase (EC 3.4.19.13)
	79	Scaffold_11	52288	50792	-	hypothetical protein
	79	Scaffold_14	1566	817	-	Regulatory protein RecX
	79	Scaffold_7	163792	162476	-	Glutamate--cysteine ligase (EC 6.3.2.2)
	79	Scaffold_8	521170	519353	-	Gamma-glutamyltranspeptidase (EC 2.3.2.2) @ Glutathione hydrolase (EC 3.4.19.13)
	92	Scaffold_1	730143	731132	+	Integral membrane protein
	92	Scaffold_1	1237732	1239519	+	Gamma-glutamyltranspeptidase (EC 2.3.2.2) @ Glutathione hydrolase (EC 3.4.19.13)
	92	Scaffold_2	757206	758054	+	Oxidoreductase
	93	Scaffold_9	555924	557159	+	Glutamate--cysteine ligase (EC 6.3.2.2)
**Carotene**	39	Scaffold_10	189321	188473	-	Fatty acid hydroxylase family (carotene hydroxylase/sterol desaturase)
	79	Scaffold_11	262245	263762	+	Beta-carotene ketolase
	79	Scaffold_17	1367166	1368101	+	Fatty acid hydroxylase family (carotene hydroxylase/sterol desaturase)
	79	Scaffold_8	58259	57318	-	Fatty acid hydroxylase family (carotene hydroxylase/sterol desaturase)
	93	Scaffold_4	48969	47398	-	Beta-carotene ketolase
**β-carotene**	79	Scaffold_11	262245	263762	+	Beta-carotene ketolase
	93	Scaffold_4	48969	47398	-	Beta-carotene ketolase
	92	Scaffold_2	550100	551683	+	PTS system, mannitol-specific IIC component / PTS system, mannitol-specific IIB component (EC 2.7.1.197)
	92	Scaffold_2	551770	552207	+	PTS system, mannitol-specific IIA component (EC 2.7.1.197)

	92	Scaffold_2	552204	553370	+	Mannitol-1-phosphate 5-dehydrogenase (EC 1.1.1.17)
	93	Scaffold_9	129184	129849	+	TRAP-type mannitol/chloroaromatic compound transport system, large permease component
	93	Scaffold_14	443210	443911	+	2-deoxyglucose-6-phosphate hydrolase (EC 3.1.3.68) @ Mannitol-1-phosphatase (EC 3.1.3.22) @ Sorbitol-6-phosphatase (EC 3.1.3.50)
	93	Scaffold_19	64380	63940	-	PTS system, mannitol-specific IIA component (EC 2.7.1.197)
	93	Scaffold_19	64681	64373	-	PTS system, mannitol-specific IIC component
	93	Scaffold_19	65907	64687	-	PTS system, mannitol-specific IIC component
**Peroxynitrite Reduction (ahpC)**	39	Scaffold_7	107589	108047	+	AhpC/TSA family protein
**Nitric oxide reductase**	39	Scaffold_11	170944	170798	-	Anaerobic nitric oxide reductase transcription regulator NorR
	39	Scaffold_12	83656	82094	-	Anaerobic nitric oxide reductase transcription regulator NorR
	79	Scaffold_12	29818	30666	+	Anaerobic nitric oxide reductase transcription regulator NorR
	92	Scaffold_1	1047151	1046801	-	Anaerobic nitric oxide reductase transcription regulator NorR
**Lipoic_acid_metabolism**	39	Scaffold_8	108169	108447	+	Proposed lipoate regulatory protein YbeD
	79	Scaffold_8	492352	493416	+	Lipoate-protein ligase A
	92	Scaffold_2	727368	726319	-	Lipoate-protein ligase A
**catalase-peroxidase (KatG)**	39	Scaffold_12	164185	166374	+	Catalase-peroxidase KatG (EC 1.11.1.21)
	39	Scaffold_5	102621	104843	+	Catalase-peroxidase KatG (EC 1.11.1.21)
	79	Scaffold_5	59143	56888	-	Catalase-peroxidase KatG (EC 1.11.1.21)
	92	Scaffold_2	489199	486857	-	Catalase-peroxidase KatG (EC 1.11.1.21)
	93	Scaffold_9	447537	449813	+	Catalase-peroxidase KatG (EC 1.11.1.21)
**Catalase (KatE)**	39	Scaffold_10	157531	158538	+	Catalase KatE (EC 1.11.1.6)
	39	Scaffold_9	65808	67250	+	Catalase KatE (EC 1.11.1.6)
	79	Scaffold_13	39539	40996	+	Catalase KatE (EC 1.11.1.6)

	79	Scaffold_14	249089	247566	-	Catalase KatE (EC 1.11.1.6)
	79	Scaffold_14	729420	727969	-	Catalase KatE (EC 1.11.1.6)
	92	Scaffold_2	243700	245355	+	Catalase KatE (EC 1.11.1.6)
	93	Scaffold_9	497461	499707	+	Catalase KatE-intracellular protease (EC 1.11.1.6)
**Superoxide Dismutase (SOD)**	39	Scaffold_12	101291	100680	-	Superoxide dismutase [Mn] (EC 1.15.1.1)
	39	Scaffold_14	141962	142543	+	Superoxide dismutase [Fe] (EC 1.15.1.1)
	79	Scaffold_14	644563	644958	+	Nickel-dependent superoxide dismutase (EC 1.15.1.1)
	79	Scaffold_17	432530	431889	-	Superoxide dismutase [Fe-Zn] (EC 1.15.1.1)
	92	Scaffold_1	683570	684190	+	Superoxide dismutase [Mn/Fe] (EC 1.15.1.1)
	93	Scaffold_14	167374	167778	+	Nickel-dependent superoxide dismutase (EC 1.15.1.1)
**Denitrification**	39	Scaffold_12	84042	85259	+	NnrS protein involved in response to NO
	39	Scaffold_12	83656	82094	-	Anaerobic nitric oxide reductase transcription regulator NorR
**Nitrosative_stress**	39	Scaffold_12	84042	85259	+	NnrS protein involved in response to NO
	79	Scaffold_14	669721	670167	+	Nitrite-sensitive transcriptional repressor NsrR

Table S3. Summary table of matches between annotated genes and antimicrobial resistance genes found within the MEGARes database.

**Mechanism**	***Pseudoalteromonas piscicida* 39**	***Streptomyces parvus* 79**	***Microbacterium* sp. 92**	***Micromonospora arenicola* 93**
Aminoglycoside-resistant_16S_ribosomal_subunit_protein	2	2	0	0
Macrolide-resistant_23S_rRNA_mutation	3	1	0	0
Aminocoumarin-resistant_DNA_topoisomerases	0	2	0	0
Aminocoumarin_efflux_pump	0	1	0	0
EF-Tu_inhibition	0	1	0	1
Fluoroquinolone-resistant_DNA_topoisomerases	0	1	1	1
Rifampin-resistant_beta-subunit_of_RNA_polymerase_RpoB	0	1	0	2
Pyrazinamide-resistant_mutant	0	0	0	1

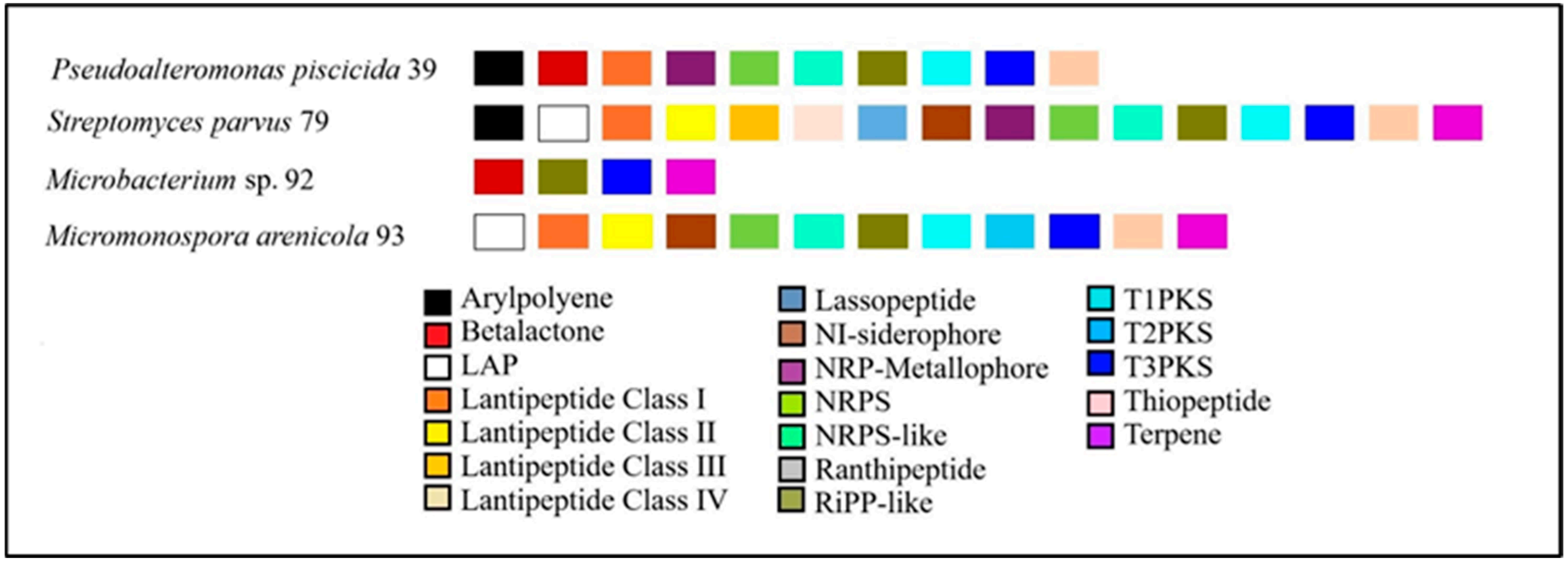

Figure S1. Graphical visualisation of relevant BCGs for natural products production present in the *P. piscicida* 39, *S. parvus* 79, *Microbacterium* sp. 92 and *M. arenicola* 93 obtained from antiSMASH analysis.

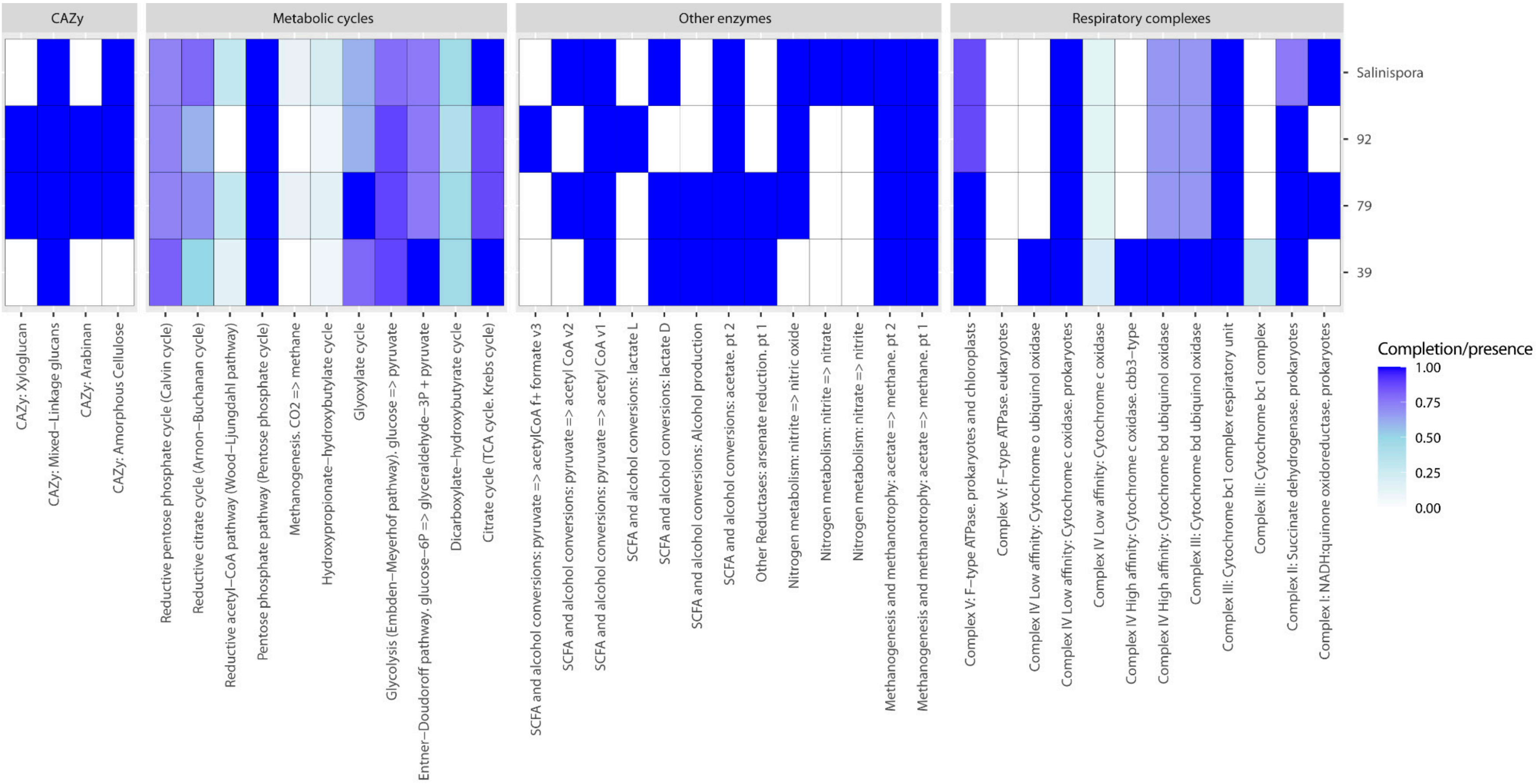

Figure S2. Heatmap representing the completion and/or presence/absence of specific metabolic cycles and enzymes within the sequenced genomes.
